# Association between a 17-gene genomic prostate score and multi-parametric prostate MRI in men with low and intermediate risk prostate cancer (PCa)

**DOI:** 10.1371/journal.pone.0185535

**Published:** 2017-10-10

**Authors:** Michael S. Leapman, Antonio C. Westphalen, Niloufar Ameli, H. Jeffrey Lawrence, Phillip G. Febbo, Matthew R. Cooperberg, Peter R. Carroll

**Affiliations:** 1 Departments of Urology, Yale University School of Medicine, New Haven, Connecticut, United States of America; 2 Department of Urology, University of California San Francisco, San Francisco, California, United States of America; 3 Department of Radiology, University of California San Francisco, San Francisco, California, United States of America; 4 Genomic Health Inc., Redwood City, California, United States of America; 5 Department of Epidemiology and Biostatistics, University of California San Francisco, San Francisco, California, United States of America; King's College London, UNITED KINGDOM

## Abstract

**Background:**

We aimed to directly compare results from multi-parametric prostate MRI (mpMRI) and a biopsy-based 17-gene RT-PCR assay providing a Genomic Prostate Score (GPS) among individuals who were candidates for active surveillance with low and intermediate risk prostate cancer (PCa).

**Patients and methods:**

We evaluated the association between GPS results (scale 0–100) and endorectal mpMRI findings in men with clinically localized PCa. MR studies were reviewed to a five-tier scale of increasing suspicion of malignancy. Mean apparent diffusion coefficient (ADC) was calculated from a single dominant lesion. Mean rank of the GPS (0–100) among MRI strata was compared with the Kruskal-Wallis test and Dunn's multiple comparison test. Spearman's correlation was performed to examine the association between mean ADC and scaled GPS.

**Results:**

Of 186 patients who received GPS testing, 100 were identified who received mpMRI. Mean GPS results differed between mpMRI categories (p = 0.001); however a broad range was observed in all mpMRI categories. Among men with biopsy Gleason pattern 3+3, mean GPS results were not significantly different among MRI groups (p = 0.179), but GPS differences were seen among MRI categories for patients with pattern 3+4 (p = 0.010). Mean ADC was weakly associated with GPS (σ = -0.151). Stromal response (p = 0.015) and cellular organization (p = 0.045) gene group scores differed significantly by MRI findings, but no differences were seen among androgen signaling or proliferation genes.

**Conclusions:**

Although a statistically significant association was observed between GPS results and MRI scores, a wide range of GPS values were observed across imaging categories suggesting that mpMRI and genomic profiling may offer non- overlapping clinical insights.

## Introduction

A majority of men diagnosed with prostate cancer (PCa) have what appears to be low and intermediate-risk disease at presentation on the basis of clinical and pathological factors[1]. Yet the performance of even best clinical prediction instruments will mischaracterize a proportion of men who harbor occult, higher grade or stage disease[2]. The ability to offer improved risk stratification among such men is therefore important as such efforts may better shape the trajectory of initial management, a decision choice increasingly defined by active surveillance (AS) versus immediate treatment[3]. To this end, both tissue-based gene expression assays and multi-parametric prostate magnetic resonance imaging (mpMRI) have received considerable attention for the potential to add predictive value beyond conventional clinical models to determine the presence of high grade or non-organ confined disease and are gaining utilization in early disease management[4–7].

Biopsy based assays reflecting the quantitative expression of genes associated with tumor aggressiveness have demonstrated predictive value for adverse pathology at radical prostatectomy (RP), as well as downstream oncologic endpoints including biochemical recurrence (BCR) and metastatic progression[8]. In validation studies, these tools have added predictive performance that exceeds conventional clinical and pathological variables[5]. Similarly, high resolution MRI examining multiple imaging parameters appears to offer anatomic and biological insights into tumor stage and grade that offer higher degrees of accuracy with regard to clinical staging, tumor localization, and the likelihood of adverse downstream events[9, 10].

It is unclear, however, whether these modalities offer congruent or independent biologic information. To date, no published studies exist that compare the directionality of these tests in the same subjects. Specifically, it is unknown whether men with adverse findings on MRI will derive further benefit from tissue-based assays; or conversely, whether MRI will add meaningful information to those who have already had tissue based testing. In this context, we sought to evaluate the association between mpMRI findings and a 17- gene GPS among men with clinically favorable PCa following initial diagnosis. We hypothesized that a strong correlation would exist between MRI findings and GPS results.

## Patients and methods

### Patient selection

Under the University of California San Francisco (UCSF) institutional review board approval, we retrospectively identified all consenting patients with low or intermediate-risk PCa who underwent a 3T endorectal coil mpMRI and a biopsy-based RT-PCR assay (Onco*type* DX^®^ Prostate Assay) providing a Genomic Prostate Score (GPS) as a measure of tumor aggressiveness within a maximum of two years between studies. Patient records were de-identified and analyzed anonymously. Among patients with initial biopsy at our institution diagnostic biopsies were performed using extended sextant systematic sampling techniques including a minimum of 12 cores; those performed at referring centers were reviewed by genitourinary pathologists to establish the Gleason score (GS) and volume of disease. Disease risk was defined using the validated Cancer of the Prostate Risk Assessment score (UCSF-CAPRA)[11].

mpMRI tests were obtained at the discretion of the providers as a local staging tool for men with early stage disease who were considering or enrolled in AS to establish disease stability. MR sequences included T2, high B-value diffusion-weighted imaging (DWI), MR spectroscopic imaging (MRSI) and dynamic contrast enhancement (DCE)[12]. Scans were acquired on a 3-Tesla scanner (GE Healthcare, Waukesha, WI) using the body coil for excitation and an endorectal coil (E-Coil, Medrad, Pittsburgh, PA) filled with perfluorocarbon and a phased-array coil for reception. Images were post-processed to compensate for the reception profile of the endorectal coil. All MRI studies were re-reviewed by a genitourinary radiologist with 10 years of experience blinded to the biopsy and GPS results and graded on a 1–5 scale of increasing suspicion of malignancy, a modification of the PI-RADS version 1 system (Table 1). The mean apparent diffusion coefficient (ADC) was calculated from a single dominant lesion[13]. The combination of MR imaging and genomic profiling was routinely recommended for men with low and intermediate clinical risk disease prior to enrollment in AS as a means of refined risk assessment.

**Table 1:** Multi-parametric prostate MRI scoring rubric.

***T2 –Peripheral Zone***			
1	Homogeneous high SI		
2	Streaky, triangular, geographic areas of low SI		
3	Not 1/2 not 3/4		
4	Discrete nodule of low SI		
5	Same as 4 + evidence of ECE and/or > 1.5 cm of capsular contact		
	***T2 –Transitional Zone***		
1	Heterogeneous SI, "organized chaos"		
2	Foci of low SI, well marginated /encapsulated		
3	Not 1/2 not 3/4		
4	Foci of homogeneous low SI, ill defined		
5	Same as 4 + evidence of ECE and/or > 1.5 cm of capsular contact		
***Diffusion Weighted Imaging (DWI)***			
	*High B value DWI*	*ADC*	*ADC Value*
1	Iso SI	Iso SI	N/A
2	High SI	Iso SI	N/A
3	Iso SI	Low SI	N/A
4	High SI	Low SI	> 850
5	High SI	Low SI	< 850
***Magnetic Resonance Spectroscopy Imaging (MRSI)***			
1	Citrate peak 2 x > choline peak		
2	Citrate peak 1–2 x > choline peak		
3	Citrate peak = choline peak		
4	Choline peak 1–2 x > citrate peak		
5	Choline peak 2 x > citrate peak		
***Dynamic Contrast Enhancement (DCE)***			
+	Asymmetric early enhancement with quick washout or plateau		
-	Other patterns of enhancement		

The OncotypeDX Prostate Cancer assay (Genomic Health, Inc., Redwood City, CA) was performed using RNA extracted from fixed paraffin-embedded diagnostic prostate needle biopsies. This biopsy-based RT-PCR assay generates a Genomic Prostate Score (GPS–scaled 0–100) as a biologic measure of tumor aggressiveness, and has been clinical validated as an independent predictor of favorable surgical pathology (surgical GS <4+3 and organ-confined disease) [5] [14]. The GPS represents a weighted calculation of a 17-gene expression signature including 12 genes highly associated with prostate cancer recurrence and metastases and five reference genes to control for RNA quality and quantity[5] [15]. The four constituent gene groups represented in the GPS include androgen signaling (AZGP1, KLK2, SRD5A2, RAM13C), stromal response (BGN, COL1A1, SFRP4), cellular organization (FLNC, GSN, TPM2, GSTM2) and proliferation (TPX2).

### Statistical analysis

Our primary objective was to evaluate the association between mpMRI findings and GPS results among an observational cohort of men with clinically localized prostate cancer; we compared the mean rank of the scaled GPS (0–100) across MRI results characterized as negative (score 1–2); indeterminate (3); or positive/suspicious (4–5) using the Kruskal-Wallis test. To examine differences among groups, we further performed a post hoc pairwise analysis using Dunn’s multiple comparison test, a method that retains the dependent ranking produced by the Kruskal-Wallis test statistic and incorporates the pooled variance estimate and also preserves the family-wise error rate by using adjusted significance level defined as α/(k(k-1)), where k is the number of comparisons[16]. To examine particular associations among individual gene groups with MR findings we repeated the Kruskal-Wallis analyses within expression scores for all gene groups. As diffusion weighted imaging (DWI) been proposed as a quantitative measurement associated with tumor aggressiveness, we further sought to examine the association between mean apparent diffusion coefficient (ADC) and GPS using Spearman’s correlation[17]. Analysis was performed among all pooled 100 patients, as well as stratified by CAPRA risk category and biopsy GS 3+3 or 3+4. Statistical analyses were performed using SAS software version 9.3 (SAS Institute, Cary, North Carolina, USA).

## Results

Among 186 consented men who have undergone GPS testing at our institution, we identified 100 with mpMRI within a two year window of genomic testing. Compared with men undergoing combined MRI and GPS testing those undergoing GPS testing alone were similarly matched with regard to baseline demographic and disease characteristics. Among patients receiving both MRI and GPS median age was 62.5 years and median PSA was 5.6 ng/mL (IQR 4.3–8.3). Biopsies included a median of 16 cores (interquartile range, IQR 13–19), and a median of three cores were positive for cancer (IQR 1–4). 53 men had biopsy GS3+3 and 47 GS 3+4. The majority of patients (n = 63) sought initial management with AS while 41 ultimately underwent treatment with radical prostatectomy. The complete clinical and pathologic features are presented in Table 2.

**Table 2:** Baseline characteristics of 100 patients receiving multi-parametric prostate MRI and GPS testing.

Patient Characteristics	Value	Statistic
Age (Years), Median (IQR)		62.5 (55.0–68.0)
PSA (ng/ml), Median (IQR)		5.6 (4.3–8.3)
Prostate volume (cm^3^), Median (IQR)		38.0 (28.0–47.0)
PSA density, Median (IQR)		0.138 (0.098–0.189)
Race/ethnicity, N (%)*	Asian/Pacific Islander	2 (2.2)
	White	80 (86.0)
	Other	11 (11.8)
Clinical T-stage, N (%)	T1c	75 (75.0)
	T2	25 (25.0)
# Cores taken, Median (IQR)		16.0 (13.0–19.0)
# Cores positive, Median (IQR)		3.0 (1.0–4.0)
% Cores positive, Median (IQR)		17.0 (8.0–25.0)
% Single core positive, Median (IQR)		28.0 (15.0–44.0)
CAPRA, N (%)*	Low (0–2)	66 (79.5)
	Intermediate (3–5)	16 (19.3)
	High (6–10)	1 (1.2)

*Abbreviations*: IQR = interquartile range; PSA = prostate-specific antigen; CAPRA = Cancer of the Prostate Risk Assessment

*Due to missing data, totals do not sum to 100 patients

**Fig 1:**
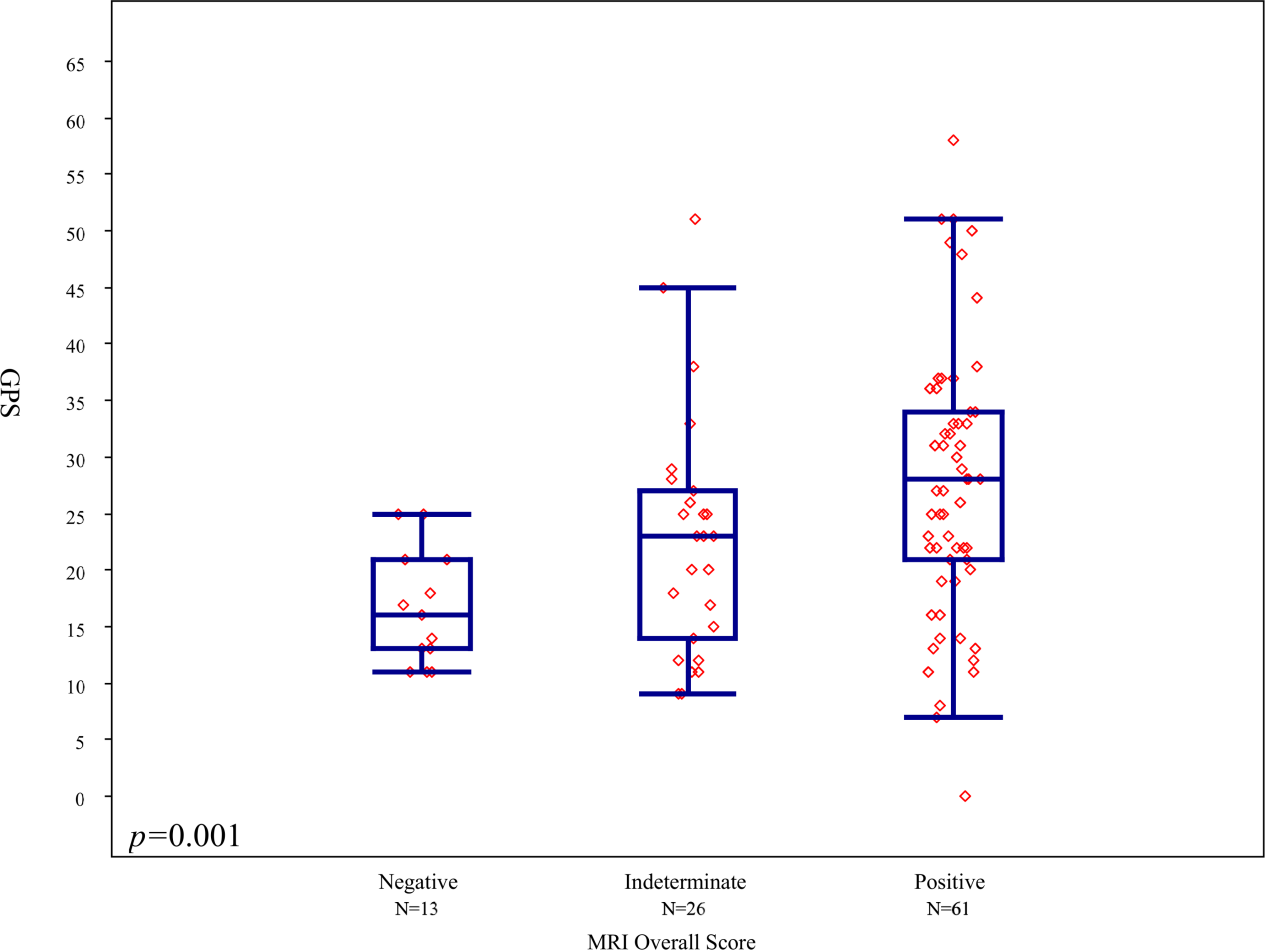
Distribution of GPS scores by MRI findings among all 100 patients.

**Fig 2:**
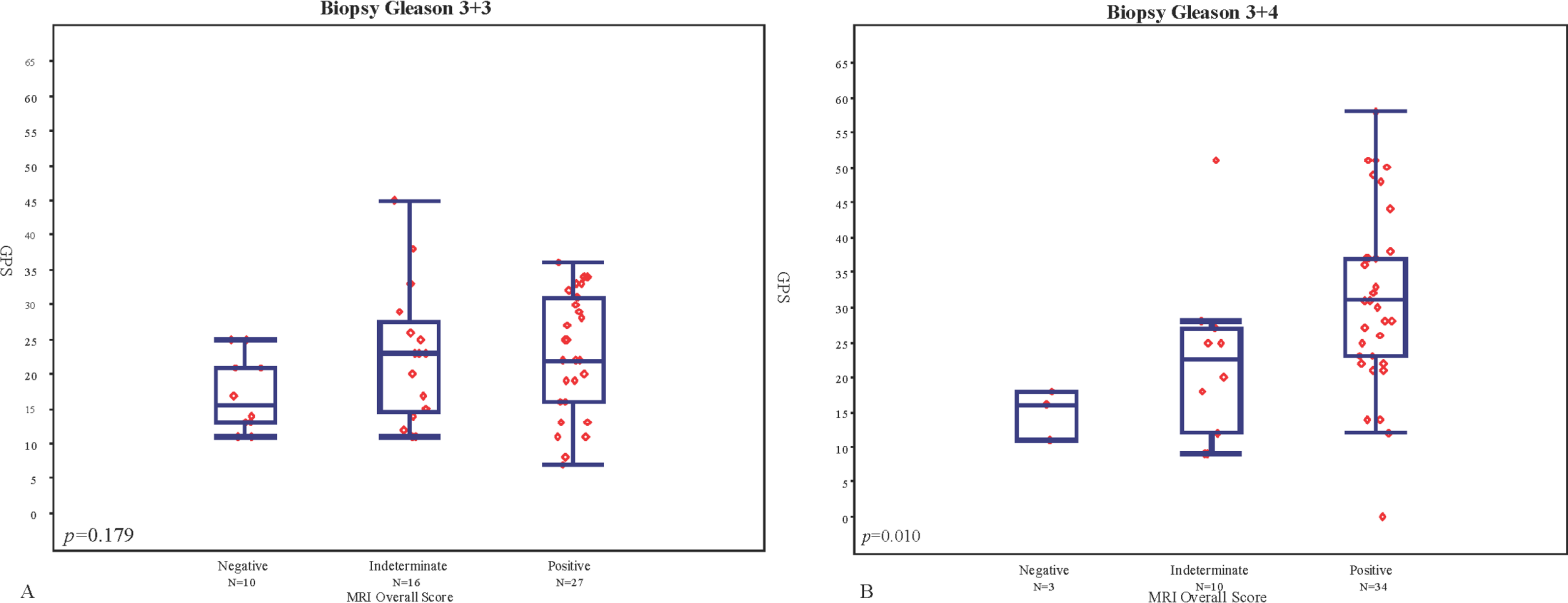
A. Distribution of GPS results by MRI findings among men with biopsy Gleason grade 3+3 and B. Among patients with Gleason grade 3+4.

**Fig 3:**
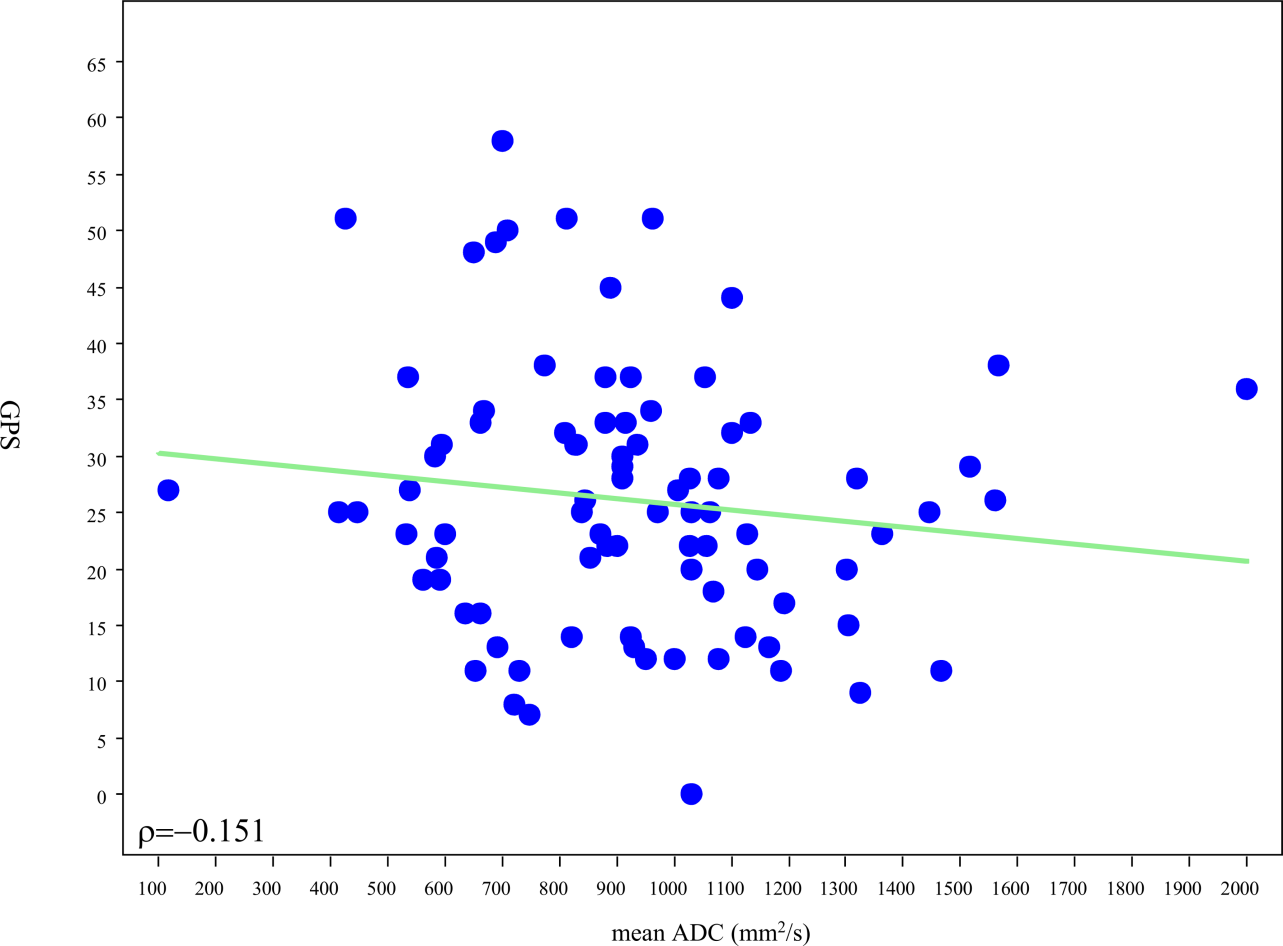
Relationship between meanADC value (mm^2^/s) of the dominant MRI lesion and GPS result.

MRI findings were negative in 13 patients, indeterminate in 26, and positive in 61. The median GPS was 16 (IQR 13–21) for men with negative, 23 (IQR 14–27) for indeterminate and 28 for positive (IQR 21–34) MRI studies, Fig 1. There was a significant difference in the mean rank of the GPS results among the 3 MRI categories (p = 0.001) for the entire group of 100 patients, with mean GPS increasing with increasing MRI category. Nonetheless there was a wide distribution of GPS values within each MRI category. When patients were stratified by biopsy GS, no significant association (p = 0.18) was observed between GPS and MRI in patients with GS 3+3 (Fig 2A), however there was a significant association among men with GS 3+4 tumors (p = 0.010 –Fig 2B). Post-hoc pairwise comparisons showed significant difference in mean rank of GPS between negative and positive MRI categories (p<0.001), however no statistically significant difference was observed between negative and indeterminate or positive and indeterminate results. Among men with high suspicion MRI lesions we evaluated the association between scaled GPS results and meanADC values. A weak trend towards higher GPS results was observed with lower meanADC values, (Rho = -0.151), (Fig 3).

**Fig 4:**
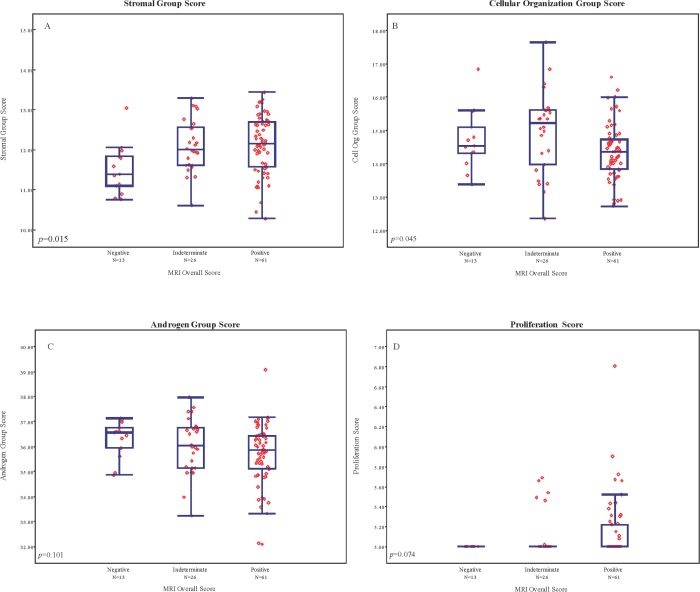
A-D. Distribution of individual gene group expression levels among mpMRI categories.

To determine whether further associations may exist within individual gene pathways measured by GPS and MRI findings, we examined the association of gene group expression levels with MRI findings. Stromal response and cellular organization gene expression scores showed a modest but statistically significant association with MRI category (p = 0.015 and p = 0.045, respectively), however no significant trends were observed in androgen response or proliferation (p = 0.101 and 0.074, respectively). meanADC also exhibited weak association with individual gene groupings (stromal response: Rho = -0.221; cellular organization Rho = -0.01; androgen signaling Rho = 0.106; and proliferation Rho = 0.01). Within each gene pathway, considerable variation in expression levels was observed among all MRI groups, particularly among the 61 patients with category 4 and 5 lesions (Fig 4A–4D).

## Discussion

The limitations of conventional clinical variables to reliably characterize the extent of disease among men with newly diagnosed PCa have been met with a growing arsenal of tools aimed to improve risk estimation in early stage disease. Genomic signatures have been validated to predict the occurrence of higher grade or stage elements among clinically favorable risk patients, while high resolution mpMRI as both an anatomic and biologically informative modality has received extensive study for the ability to detect significant cancer[4, 5, 18]. Comparative analyses of such advanced risk stratification tools, however, are lacking and it remains unclear whether individuals who have received MR imaging will derive further benefit from genomic testing, or conversely, if imaging may be of benefit among men following tissue-based assays.

In this study, we examined the association between MRI findings and the GPS signature in a cohort of 100 men with clinically favorable risk PCa undergoing evaluation with both modalities. Although a trend towards higher GPS results existed among higher MR-suspicion lesions, we observed considerable variation across all mpMRI findings, suggesting biologic heterogeneity within each of the MRI categories. Among men without MR-evident tumors, GPS results tended to be lower and more narrowly distributed, however this represented a minority of patients in the study (13%). For men with indeterminate or positive MR studies, GPS results ranged significantly, a relationship that persisted after stratification by GS or clinical risk group. Though not directly correlated with patient outcome, these findings suggest that further clinical refinement may be possible among patients beyond MRI findings alone.

The GPS assay is derived from a set of highly predictive genes in a fashion independent of Gleason pattern, and has been validated by independent studies as a predictor of pathological upgrading and/or upstaging among men with favorable risk disease [5] [14]. It has also been shown to predict distant oncological outcomes, including biochemical recurrence and metastatic progression[5, 14]. We speculated that imaging findings would recapitulate tumor gene expression levels: higher suspicion MRI lesions would be associated with higher GPS scores, higher expression levels of stromal response and proliferation genes would be associated with MR-evident disease, and that areas of restricted diffusion would be associated with higher GPS findings. We observed that GPS results were significantly different among patients with negative, indeterminate, or positive MRI scans, and among men with biopsy pattern 3+4, though no significant differences were seen among MRI groups for men with biopsy pattern 3+3. Moreover, stromal response and cellular organization gene expression scores were associated with MR findings, though no associations were seen among androgen signaling or proliferation groups. As prior clinical validation studies have demonstrated significant associations of the four primary gene groupings with clinical outcome, including recurrence, these finding may indicate a novel radiomic basis for stromal response and cellular organization genes and MR pattern. Taken together these findings suggest that a degree of agreement in signal direction exists between MRI and the GPS assay, however the information provided by these two modalities appears to be largely non-overlapping.

The prognostic significance of a visible lesion on mpMRI has been evaluated on several PCa endpoints including the detection of higher grade (Gleason ≥3+4) disease on biopsy[19], progression during AS, high grade/stage pathology at prostatectomy[20], and biochemical recurrence[21]. For men with newly diagnosed PCa, the presence of MRI-discernable lesions has been suggested as an adverse prognostic characteristic particularly for men initiating management with AS, where retrospective studies have suggested an increased risk for disease reclassification due to changes in tumor volume estimates[22]. As MRI studies incorporate multiple sequences believed to reflect anatomic, tumor vascularity, and cellularity it is not known whether higher rates of upgrading reflect improved sampling associated with larger tumors in some men or the occurrence of genuine biological progression.

Though MRI has exhibited the capacity to identify higher Gleason pattern PCa, the genetic mechanisms underlying characteristic MRI findings remain to be fully elucidated. In this study mean, ADC values exhibited weak, negative correlation with overall GPS or constituent gene groups. These findings are notable given prior studies addressing the association of apparent diffusion coefficient (ADC) analysis and prostate cancer aggressiveness[17]. The directionality of the relationship is consistent with the implication of restricted diffusion with tumor aggressiveness, however the strength of the association appears to imply that meanADC analysis does not closely parallel a panel of genes highly associated with PCa aggressiveness.

We included biopsy samples obtained using systematic extended sextant sampling and not direct MR-ultrasound fusion acquisition, reflecting the era of collection at our institution. As a result, we were not able to compare gene expression profiles on a per-lesion basis. In addition, because of a growing appreciation for improved diagnostic yield associated with MR-ultrasound fusion, it is conceivable that some degree of undersampling may be present relative to a targeted method[18] [23]. As the GPS assay has been previously shown to predict prostate cancer aggressiveness in the face of biopsy under-sampling and heterogeneity in Gleason grade, our approach may hold validity in assessing the broader tumor profile of the gland, though further study is warranted using image-guidance or whole-mount correlation[5]. In this regard, important insights appear to be offered by intriguiging preliminary directed radiomic study matching gene expression to gene expression features. For example, in an analysis of 17 mpMRI-directed diagnostic biopsies, associations were observed between radiographic abnormalities and aberrant gene expression, highlighting a measurable molecular basis for characteristic MR findings [24].

An acknowledged limitation of this study is that our comparison is restricted to the two modalities themselves without direct evaluation of clinical outcomes. This finding reflects the favorable risk nature of the study cohort and the routine use of AS for initial management in appropriate candidates. As a result, only a small proportion received immediate definitive treatment with prostatectomy, which limited our ability to offer a pathological comparison. Additionally, although patients receiving genomic testing with or without MRI were evenly matched, we cannot conclude with certainty that the decision to pursue both MRI and GPS testing in these patients occurred at random. While individuals may opt for multiple modalities of advanced testing due to preference, it is possible that such patients possessed unrecognized clinical complexity which warranted additional evaluation. In addition, all MR images were reviewed by a single radiologist, which may limit reproducibility. We utilized a two-year interval between MRI and GPS testing as inclusion criteria under the assumption of disease stability during this time based on modeling and observational studies, however we cannot exclude that changes in tumor biology may have occurred during this time period[25, 26]. Lastly, the study design did not examine GPS results from biopsies of selected MRI abnormalitites. Given the growing utilization of MRI-ultrasound fusion platforms affording directed sampling of MR-apparent lesions, this limitations prevented us from comparing in genomic profiles based on direct biopsy.

Both tissue based genomic profiling and MRI seek to offer refined clinical staging and risk stratification at the time of diagnosis, including the risk of adverse pathology at the time of surgery. A host of publications support the use of MRI to predict clinical stage, and presence for extraprostatic extension or seminal vesicle invasion [27]. Similarly, the GPS assay has been clinically validated to predict pathologic outcomes among two studies including 732 patients receiving radical prostatectomy[28]. As patients in this study received imaging and genomic profiling in the clinical context of establishing eligibility for AS, a minority (n = 41) underwent treatment with prostatectomy at last follow. As a resut, direct comparisons are limited due to sample size, and selection bias as patients often receive treatment in the context of biopsy upgrade or concern for progression. To optimally detect significant differences in gene expression across five strata of MRI findings corresponding to all PI-RADS classifications, we estimate that over 300 patients would be required. Further, appropriately powered studies are required to assess the relative clinical utility of imaging and genomic profiling in initial risk stratification.

Few other studies have directly compared associations among a new generation of PCa risk prediction tools. Recently, Renard-Penna et al. examined 106 patients treated with RP in whom pre-operative mpMRI findings were compared with genomic testing of tumor tissue with a 31-gene cell progression assay as a measure of aggressiveness, noting associations among tumor size and diffusion abnormalities with adverse cell-cycle progression (CCP) scores[29]. Interestingly, the authors utilized CCP as a benchmark for assessing the performance of preoperative imaging, however, a valuable opportunity remains to compare these modalities, particularly in the pre-treatment setting. As the inventory for such risk refinement aids expands in size and complexity, it is likely that such direct comparisons will be of increasing clinical impact and potentially serve to more efficiently direct resources. Our findings suggest that mpMRI and the 17-gene GPS assay offer predictive information that may be distinctive in many circumstances reflecting intrinsic differences between these tools. Prostate MR scoring systems, including the PI-RADS framework, have been developed and calibrated to detect clinically significant cancer apprised largely on the basis of Gleason score, a powerful though altogether incomplete predictor of disease outcome[30]. In contrast, the genes selected for the GPS assay and their relative weights within the generated score were validated to predict adverse pathologic and oncological events independently of pathological assessment. As a result, we anticipated that heterogeneity in gene expression levels would exist within MRI groups, a finding which underscores the clinical variability within MRI groups.

The intersection of multiple PCa risk refinement tools in early stage PCa is a presently unexplored avenue of investigation with implications for disease management. Our findings, reflecting one commercial assay, demonstrate stratification among MRI findings, yet are, alone, inadequate to dictate definitive clinical sequencing of these tests. Additional comparative studies of imaging and serum detection-oriented biomarkers may also prove fruitful in clarifying optimal diagnostic pathways. Additionally, larger study populations assessing downstream PCa outcomes will be required to assess the optimal sequencing of such emerging modalities.

## Conclusion

We compared mpMRI and tissue based gene expression testing among low and intermediate clinical risk patients. Prostate MRI and genomic testing with the GPS assay exhibited weak correlation, suggesting that the two modalities represent independent predictors and may be complementary in guiding patient management.
